# A Systematic Review of Anogenital Distance and Gynecological Disorders: Endometriosis and Polycystic Ovary Syndrome

**DOI:** 10.3389/fendo.2021.696879

**Published:** 2021-07-22

**Authors:** Zhenyan Pan, Fangfang Zhu, Kai Zhou

**Affiliations:** Department of Gynecology, The First Affiliated Hospital of Wenzhou Medical University, Wenzhou, China

**Keywords:** androgen, anogenital distance, endometriosis, polycystic ovary syndrome, prenatal exposure, reproduction

## Abstract

**Background and Aim:**

Anogenital distance (AGD) can serve as a life-long indicator of androgen action in gestational weeks 8–14. AGD has been used as an important tool to investigate the exposure to endocrine-disrupting compounds in newborns and in individuals with male reproductive disorder. Endometriosis and polycystic ovary syndrome (PCOS) are two common gynecological disorders and both are related to prenatal androgen levels. Therefore, we performed a systematic review to evaluate the relationships of AGD with these gynecological disorders.

**Methods:**

PubMed, Web of Science, and Embase were searched for published studies up to January 25, 2021. No language restriction was implemented.

**Results:**

Ten studies were included in this review. Five focused on women with endometriosis, and six investigated women with PCOS. According to these studies, PCOS patients had longer AGD than controls, while endometriosis patients had shorter AGD than controls. In conclusion, this study provides a detailed and accurate review of the associations of AGD with endometriosis and PCOS.

**Conclusion:**

The current findings indicate the longer AGD was related to PCOS and shorter AGD was related to endometriosis. However, further well-designed studies are needed to corroborate the current findings.

## Introduction

Anogenital distance (AGD) is defined as the distance between the anus and genital tubercle in fetuses. AGDAC and AGDAF are defined as the distance from the anterior clitoral surface or posterior fourchette, respectively, to the upper/center verge of the anus. Initially, the development of external genitalia in mammals is morphologically indistinguishable between male (XY) and female (XX) fetuses. The later differences depend on whether the Y-chromosomal gene *Sry* is expressed. Testes develop in male fetuses, which causes testosterone to be produced by Leydig cells. The only testosterone in female fetuses comes from the fetal adrenal glands and the maternal adrenal glands, ovaries, and adipose tissue. Androgen affects the development of the perineal tissue, increasing the distance between the anus and genital tubercle (1). Therefore, AGD can serve as a life-long indicator of androgen action in gestational weeks 8–14, which is known as the masculinization programming window (MPW) (2). Generally, boys have a longer AGD than girls, with an ~2-fold difference up to 24–30 months of age. Although there is a lack of statistical evidence on this male–female difference before and during adolescence, the difference has been shown to be maintained throughout adulthood. The difference has been observed in first-trimester fetuses by transabdominal ultrasound, and a shorter AGD in male fetuses indicates diseases associated with testicular dysplasia syndrome (3–5). According to rodent models and human data, the AGD of an individual changes as their body grows (6).

AGD has been used as an important tool for investigating exposure to endocrine-disrupting compounds in newborns and in individuals with male reproductive disorders. In male fetuses, AGD is regulated by dihydrotestosterone activation of androgen receptors. However, the regulatory mechanism in female fetuses is unclear; in addition to the influence of androgen receptors, the mechanism may be related to estrogen and progesterone (1). AGD is associated with several congenital disorders of male reproductive development (such as cryptorchidism and hypospadias) and other disorders that emerge in adulthood (such as low sperm count and prostate cancer) (7–9). The effectiveness of AGD as a biomarker or predictor of disease in females has not yet been confirmed, but several recent studies have provided strong evidence for this.

Endometriosis is a common benign gynecological disorder that is characterized by chronic pelvic pain, dysmenorrhea, and/or infertility. It affects ~5–10% of women in their reproductive years. It is defined as the growth of functional endometrium-like tissues outside of the uterine cavity (especially in the pelvic cavity), affecting the pelvic organs, uterosacral ligaments, pouch of Douglas, and distant organs such as the lungs and brain (10). The most accurate diagnostic method for endometriosis is laparoscopy with histology. However, the requirement for invasive diagnostic methods and/or the relatively highly prevalent but nonspecific clinical symptoms delays diagnosis. According to a multicenter study, there was a mean delay of 6.7 years, leading to financial loss and psychological distress (11). Transvaginal ultrasonography along with taking a medical history is the first-line approach for investigating pelvic endometriosis in the clinic. However, this relies on the skill of the sonographer and is unreliable if the peritoneal endometriosis lesions are small (12). Endometriosis is a hormone-dependent disorder, and exposure to estrogen and anti-androgen endocrine disruptors have been reported to significantly increase the risk of its occurrence. Endometriosis may be related to relatively low prenatal and postnatal testosterone levels, which may disrupt hypothalamic–pituitary–ovarian (HPO) axis development (13). Moreover, it has recently been hypothesized that decreased AGD is linked to endometriosis *via* the gut–genital microbiota and resultant subclinical infections (14). Adding AGD to the endometriosis diagnostic strategy may help to shorten the diagnostic delay.

Polycystic ovary syndrome (PCOS) is the most common endocrine disorder in reproductive-aged women, affecting 5–20% of women worldwide. The condition is characterized by hyperandrogenism, ovulatory dysfunction, and polycystic ovary morphology (15). PCOS is a complex disorder caused by a variety of environmental and genetic factors including high prenatal androgen exposure. Animal models have shown that prenatal androgenization can produce PCOS-like phenotypes, which include excessive luteinizing hormone (LH) and metabolic abnormalities, along with hyperandrogenism, oligoovulation, and polyfollicular ovaries (16, 17). It has also been clinically observed that women with congenital adrenal hyperplasia are more likely to experience PCOS during adulthood, despite treatments that normalize androgen excess after birth (18).

Hormones represent common risk factors for both endometriosis and PCOS, but their hormonal characteristics are very different. Endometriosis patients had lower LH relative to follicle-stimulating hormone (FSH), higher sex hormone-binding globulin (SHBG), higher serum oxytocin, lower serum testosterone, and lower anti-Müllerian hormone (AMH). However, PCOS patients had higher LH relative to FSH, lower SHBG, lower serum oxytocin, higher serum testosterone, and higher AMH. These differences indicate that the two disorders may have opposite causes, as is the case with osteoporosis and osteoarthritis, preeclampsia and postpartum hemorrhage, and cancer and neurodegeneration (13). Moreover, in PCOS patients, a longer AGD is associated with higher prenatal androgen exposure, and endometriosis patients have been reported to have a shorter AGD than controls, raising the possibility that prenatal androgen levels are related to the development of both PCOS and endometriosis.

In summary, endometriosis and PCOS are two common gynecological disorders that may be influenced by hormone levels. Recent studies have investigated whether AGD is a clinical biomarker of endometriosis and/or PCOS. We systematically reviewed published studies on the associations of AGD with these gynecological disorders in order to synthesize the results.

## Methods

### Search Strategy

A systematic review of studies was performed by conducting an electronic search of the literature published up to January 25, 2021 in PubMed, Web of Science, and Embase. In PubMed, the search strategy was: ((anogenital distance[Title/Abstract]) OR (AGD[Title/Abstract])) OR (anal genital distance[Title/Abstract]). In Web of Science, the search strategy was: (anogenital distance or AGD or anal genital distance) using a topic search. In Embase, the search strategy was: ‘anogenital distance’:ab,ti OR agd:ab,ti OR ‘anal genital distance’:ab,ti. The search results were downloaded to EndNote X9.3.2 (Thomson Corporation Corp, Stanford, CT, USA) to merge the references and remove duplicates.

### Study Selection

Article screening and eligibility evaluation was conducted following the 2009 Preferred Reporting Items for Systematic Reviews and Meta-Analyses (PRISMA) flow diagram. At the first level of screening (based on the title and abstract), we included all human studies that investigated gynecological disorders (i.e., endometriosis and PCOS) that have been reported to be related to AGD. We obtained the full-text articles of all titles that satisfied these inclusion criteria. At the second level of screening, we included only original empirical research studies that evaluated whether AGD was associated with endometriosis and/or PCOS in adult females. 

### Data Extraction

We extracted the following information from each study: first author, year, country, study design, number of patients enrolled, AGDAC and AGDAF (distance from anterior clitoral surface or posterior fourchette, respectively, to upper/center verge of the anus, in mm), and results. No statistical quantitative meta-analysis was performed due to study heterogeneity.

## Results

**Figure 1:**
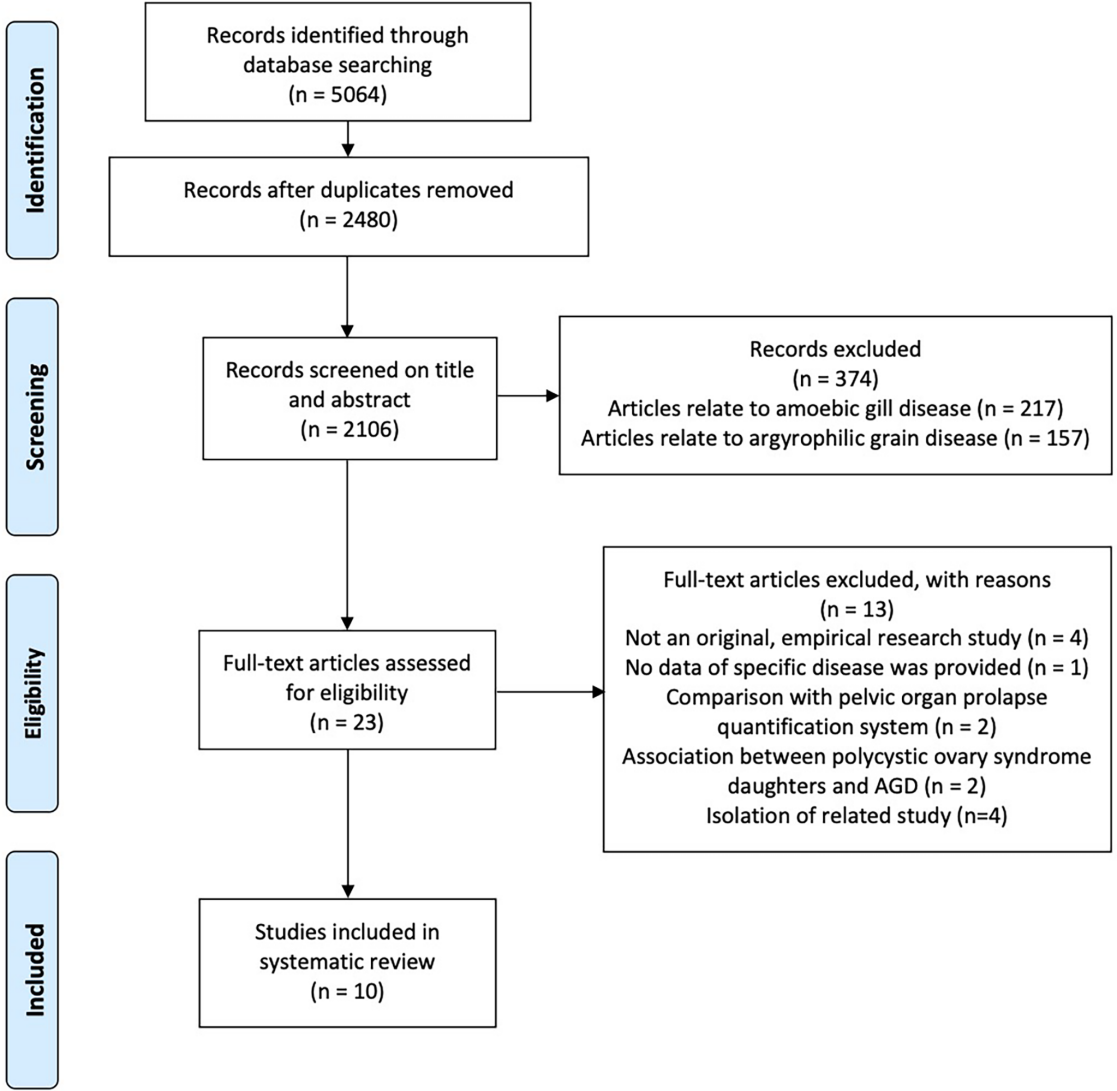
PRISMA 2009 flow diagram.

The search resulted in a total of 5,064 journal articles. After we removed the duplicates, 2,480 potentially relevant articles remained. Of these, 374 were excluded based on the title and abstract, leaving 23 that were assessed for eligibility based on the full-text articles. Ten studies met the eligibility criteria. Hence, these studies were included in this systematic review (**Figure 1**). Five focused on women with endometriosis, and six investigated women with PCOS.

### AGD Measurements

The included studies measured AGDAC (distance from anterior clitoral surface to upper/center verge of the anus) and/or AGDAF (distance from posterior fourchette to upper/center verge of the anus).

### Association of AGD With Endometriosis

Three studies were conducted by a single research group in Spain. In these studies, endometriosis diagnosis was based on medical history and transvaginal ultrasound, and patients were divided into endometrioma and deep infiltration endometriosis (DIE) subgroups (19, 20) (**Table 1**, **Supplementary Material**). In the endometrioma subgroup, AGDAF was significantly decreased compared to in healthy controls. In the DIE subgroup, both AGDAF and AGDAC were significantly decreased compared to in healthy controls. Thus, in general, decreased AGDAF was associated with endometriosis. Regardless of whether the analysis was adjusted for age and BMI or age, BMI, vaginal delivery, and episiotomy, AGDAF was more strongly associated with DIE than endometriomas. In terms of diagnostic accuracy, AGDAF was a better indicator than AGDAC, especially for DIE. Regarding DIE diagnosis, the area under the receiver operating characteristic curve (AUC) of AGDAF (with an optimal cut-off of 20.9 mm) was 0.91, achieving a sensitivity of 84.4% and a specificity of 91.4%.

**Table 1:** Studies evaluating anogenital distance in women with endometriosis.

Author	Year	Country	Study design	Number of patients enrolled	AGDAC and AGDAF (mm)	Outcome
Mendiola et al. (19)	2016	Spain	case–control	cases n = 114 controls n = 105	AGDAC: Cases: 73.8 (12.1) Controls: 75.7 (11.7) AGDAF: Cases: 23.5 (5.8) Controls: 27.3 (5.7)	Shorter AGD is associated with presence of endometriomas and deep infiltration endometriosis.
Sánchez-Ferrer et al. (20, 21)	2017					
Sánchez-Ferrer et al. (22)	2019	Spain	case–control	cases n = 57 controls n = 93	AGDAF: Cases: 22.8 (4.6) Controls: 27.2 (5.7)	Women in the endometriosis group had significantly shorter AGDAF compared with the control group.
Crestani et al. (23)	2020	France	prospective cohort	cases n = 98 controls n = 70	AGDAC: Cases: 83.8 (12.9) Controls: 100.9 (20.6) AGDAF: Cases: 21.5 (6.4) Controls: 32.3 (8.1)	Surgically and histologically proven endometriosis is associated with a short AGD in women of reproductive age but not correlated either to the severity or to the location of the disease.
Peters et al. (24)	2020	the Netherlands	case–control	cases n = 43 controls n = 43	AGDAC: Cases: 103.9 (12.6) Controls: 111.4 (13.7) AGDAF: Cases: 21.9 (6.2) Controls: 21.7 (6.2)	The AGDAC was significantly different between groups, with a decreased AGDAC in women with endometriosis.

Another Spanish study by the same research group found that endometriosis patients had significantly decreased AGDAF compared to healthy controls. Due to the small sample size of DIE patients, the study only discussed the diagnostic accuracy of AGDAF in endometrioma, which was relatively poor even in combination with AMH (22).

Crestani et al. conducted a study in France that diagnosed endometriosis patients using laparoscopy and histology. They found that endometriosis patients had a significantly decreased AGDAF compared to non-endometriosis controls. There were no differences in mean AGDAC or AGDAF between endometriosis patients with and without endometriomas or DIE. Mean AGDAC or AGDAF were not significantly different between patients with different revised American Society for Reproductive Medicine (r-ASRM) endometriosis classifications or Enzian scores. Mean AGDAC and AGDAF were also not associated with endometriosis severity or site of occurrence (23). As a diagnostic indicator of endometriosis, the accuracy of AGDAF (at a cut-off of 20 mm) was higher (AUC of 0.84, with a specificity and sensitivity of 98.6 and 30.6%) than that of AGDAC (AUC of 0.756).

In 2020, Peters et al. conducted a study in the Netherlands that included only endometriosis patients with DIE or r-ASRM ≥3. They found that AGDAC was reduced in these endometriosis patients compared to healthy controls, regardless of adjustment for BMI and age (24).

### Association of AGD With PCOS

The inclusion criteria for the PCOS patients in the following studies were mainly based on the Rotterdam criteria (25).

In 2017, Wu et al. conducted a study in China and found that AGDAF and AGDAC were longer in PCOS patients than healthy controls, and AGDAF was more significantly associated with PCOS than AGDAC (26) (**Table 2**, **Supplementary Material**). Patients who had undergone vaginal delivery were excluded from the study, and adjustments for age and BMI were conducted. Moreover, in PCOS patients, higher testosterone was positively associated with longer AGDAF and AGDAC. High LH and the presence of polycystic ovaries were also positively associated with longer AGDAF. Furthermore, in the healthy controls, there was also an association between testosterone and AGDAF.

**Table 2:** Studies evaluating anogenital distance in women with polycystic ovary syndrome.

Author	Year	Country	Study design	Number of patients enrolled	AGDAC and AGDAF (mm)	Outcome
Wu et al. (26)	2017	China	case–control	cases n = 156 controls n = 180	AGDAC: Cases: 104.9 (9.1) Controls: 97.1 (9.4) AGDAF: Cases: 26.6 (4.0) Controls: 22.0 (3.7)	The presence of PCOS is associated with longer AGD.
Sánchez-Ferrer et al. (20, 21)	2017	Spain	case–control	cases n = 126 controls n = 159	AGDAC: Cases: 80.5 (11.3) Controls: 76.0 (10.4) AGDAF: Cases: 27.8 (5.7) Controls: 26.5 (5.1)	Women with PCOS had significantly longer AGDAC and AGDAF compared to controls.
Hernández-Pen˜alver et al. (27)	2018					
Prieto-Sánchez et al. (28)	2020					
Simsir et al. (29)	2019	Turkey	prospective cohort	cases n = 65 controls n = 65	AGDAC: Cases: 101.0 (12.0) Controls: 98.0 (17.0) AGDAF: Cases: 23.0 (6.0) Controls: 21.0 (5.0)	AGD in adult PCOS patients was longer than control PCOS patients.
Peters et al. (24)	2020	the Netherlands	case–control	cases n = 43 controls n = 43	AGDAC: Cases: 113.8 (16.9) Controls: 111.4 (13.7) AGDAF: Cases: 22.0 (5.8) Controls: 21.7 (6.2)	The AGDAC was significantly different between groups, with an increased AGDAC in women with PCOS.

The Spanish team working on endometriosis also conducted three studies related to PCOS (21, 27, 28). The endometriosis groups included patients who had undergone vaginal delivery and/or episiotomy. AGDAC and AGDAF were longer in PCOS patients than healthy controls, but after adjusting for BMI, age, and episiotomy, only AGDAC remained significant. AGDAC was associated with all PCOS phenotypes and with severity. The AUC for AGDAC (using the optimal cut-off of 81.9 mm) for all PCOS phenotypes was 0.61, achieving a poor sensitivity and specificity even after adding AMH (high AMH is helpful in the identification of PCOS because it can be considered an unbiased marker of polycystic ovaries).

Simsir et al. conducted a study in Turkey and found that AGDAC and AGDAF were longer in PCOS patients than healthy controls, but not significantly (29). The study indicated that it was more meaningful to use the ratio of AGDAC to AGDAF.

The study conducted by Peters et al. in the Netherlands found that AGDAC was longer in PCOS patients than healthy controls in the unadjusted analysis, but there was no significant difference after adjusting for BMI and age (24). In the PCOS group, a significant positive association was found between biochemical hyperandrogenemia and AGDAC, but not AGDAF.

## Discussion

This systematic review provides a summary of the literature assessing the associations of AGD with gynecological disorders. To our knowledge, the only other review on this relationship is a narrative literature review by Buggio et al. (30). However, several recent studies have reported evidence that supports the hypothesized associations of AGD with gynecological disorders, which prompted us to summarize the findings in a systematic review.

In the included studies, although there was no significance in some studies and the significance of the two AGD measurements were inconsistent in many studies, it was generally found that the AGD of PCOS patients was longer than that in controls, while the AGD of endometriosis patients was shorter compared to that in controls. There are several sample-specific limitations with these studies that include, but are not limited to the small sample size, representation of the cases to reflect a true patient population and different data processing. The Spanish studies included women who had given birth vaginally and/or had an episiotomy (19–22, 27, 28). The data from the Turkish study by Simsir et al. was not adjusted for BMI, even though the BMI was significantly different between the PCOS and control groups (29). Only the endometriosis patients in the study by Crestani et al. were comprehensively diagnosed using laparoscopy and histology (23). Therefore, we believe that more studies are needed to exclude the effects of vaginal delivery and episiotomy, and to adjust for age, BMI or other body measurements.

Although the causes of endometriosis remain largely unknown, it seems to have a multifactor etiology, involving oxidative stress, inflammatory factors and cytokines, genetics, and hormones (31). The development of the female reproductive system begins with a sex difference in the expression of the *Sry* gene, followed by the development of the HPO axis, at which point the influence of prenatal androgens is important. The abnormal hormones in PCOS patients suggest that changes in the HPO axis are induced by relatively high prenatal testosterone. However, the opposite hormonal changes in endometriosis patients compared to PCOS patients indicate that relatively low prenatal androgen levels are involved in endometriosis.

The breakdown of endometrium during menstruation is a highly inflammatory process, and high estradiol and low testosterone increase the likelihood of widespread inflammation in ectopic endometrial cells. The more extreme menstrual characteristics in endometriosis (especially regarding menstrual volume) are thought to increase retrograde flow. Additionally, when prenatal testosterone is low, migration and differentiation of Müller stem cells may occur more easily (13). These findings reinforce the two major hypotheses related to endometriosis. The first hypothesis involves the reflux of endometrial cells into the peritoneal cavity during menstruation, which was proposed by Sampson. The second hypothesis involves the ectopic endometrial cells being derived from the migration and differentiation of Müllerian duct cells during early uterine development (32, 33).

In addition, increasing numbers of studies suggest that the three main phenotypes of endometriosis, namely, ovarian, peritoneal, and DIE, should be considered as different diseases, because the gene expression in these ectopic endometrial tissues varies greatly, which may indicate different etiological mechanisms. *HOX* genes are responsible for assigning identity to undifferentiated tissues and they exhibit a well-organized spatiotemporal expression pattern during embryogenesis and adulthood. Specifically, *HOXA10* regulates the conversion from embryonic to endometrial tissue. *HOXA10* was abnormally expressed in ectopic endometrial tissues and had different expression patterns in different phenotypes of endometriosis. Estrogen, progesterone, and testosterone are known modulators of *HOXA10* levels. *HOXA10*, estrogen receptor α, and progesterone receptor are highly expressed in rectosigmoid endometriosis lesions, which are characteristic lesions of DIE. In contrast, these factors are downregulated in the ectopic endometrial tissues in the ovaries and peritoneum (34).

Through endometrial biopsies, the expression of *HOXA10* in PCOS patients was downregulated. *HOXA10* expression was inhibited by testosterone **in vitro**, which also prevented the abovementioned *HOXA10* upregulation induced by estradiol or progesterone. However, previous studies regarded *HOXA10* downregulation as endometrial dysfunction, leading to reduced endometrial receptivity and reduced reproductive potential in PCOS patients (35). Knocking out *HOXA10* in male mice caused cryptorchidism, and a similar *HOXA10* deletion was found in a sample of patients with cryptorchidism, albeit in a very small sample (36, 37). Patients with cryptorchidism had shorter AGD and cryptorchidism was associated with low prenatal androgen exposure. This suggested that *HOXA10* may be involved in the pathogenesis of endometriosis and PCOS induced by prenatal androgen levels, increasing the possibility that AGD can be used as a predictive and diagnostic indicator for both disorders.

Our systematic review results support the hypothesis that a shortened AGD increases the risk of fecal microbial contamination of the vulva and vagina, resulting in a cervicovaginal microbial imbalance, a subclinical inflammatory response, and then endometriosis (14). Khan et al. found elevated levels of *Escherichia coli* in the menstrual blood of endometriosis patients, suggesting that elevated endotoxin in the peritoneal fluid may promote endometriosis progression *via* Toll-like receptor (TLR)-4 (38). None of these factors have been definitively shown to have a cause–effect relationship with endometriosis, so it is reasonable to explore additional potential risk factors.

The exact changes in AGD over a woman’s lifetime are unknown, due to the lack of data on pubertal measurements (6). However, based on the available data, we can conclude that AGD remains stable during the menstrual cycle and before and after natural pregnancy (with the exception of vaginal delivery and/or episiotomy) during reproductive age, and then decreases somewhat after menopause (39–41). In some studies of AGD in newborns and adults, the data were adjusted for physical measures such as height, weight, and BMI to control for potential confounding.

Hyperandrogenism is persistent in some types of PCOS. Both the late-trimester fetuses and newborns of women with PCOS had longer AGD, due to the high prenatal androgen exposure (42, 43). Animal models showed that diethylstilbestrol (synthetic estrogen) administration in adult male rats reversibly reduced the AGD by ~11%, while castrated rats exhibited an irreversible reduction of AGD by ~17% (44). Male rats with gonadal impairment at birth followed by testosterone treatment exhibited elongated AGD (45). Accordingly, the testosterone level was positively associated with AGD in adult women in the included studies conducted by Wu et al., though this was not confirmed by the included study conducted by Peters et al. and the Spanish study group (21, 24, 26). AGD may be affected not only by intrauterine androgen exposure but also by postnatal hormones, i.e., prenatal androgen exposure may initially dictate the AGD, but postnatal androgen exposure may be required for AGD to grow to the preplanned size (46).

Hyperandrogenism is also a diagnostic criterion for PCOS in adolescents (47). Genotype–phenotype correlation studies in both Han Chinese and European PCOS patients demonstrated that *DENND1A* was a risk allele for androgen excess (48, 49). Although androgen is produced by the ovaries and adrenal glands in women, the hyperandrogenism observed in PCOS is mainly due to enhanced androgen synthesis by follicular theca cells (15). Thus, the longer AGD in PCOS patients may also be affected by the persistent influence of androgens after puberty.

In addition to endometriosis and PCOS, other disorders and characteristics of the female reproductive system were mentioned in previous studies. Sánchez-Ferrer et al. compared the diagnostic use of AGD and Pelvic Organ Prolapse Questionnaire scores for identifying pelvic organ prolapse (50). Other researchers described the relationships between AGD and female reproductive health in terms of ovarian follicle number, ovarian response after controlled ovarian stimulation for *in vitro* fertilization, and outcomes of infertility treatment (51–53).

The current most urgent task is to measure the AGD of a large number of adolescent females of different ethnicities in order to establish a baseline for AGD in the general population. Additionally, determining which body measurements should be adjusted for in the comparison of patient groups and healthy controls and which developmental period AGD should be measured in to optimize its diagnostic value would be highly useful. Wainstock et al. conducted a 17-year prospective study and published it in 2019, but they only found that women with short AGD were more likely to develop gynecological disorders without specific disease (54). Further prospective studies on gynecological disorders are underway. Additionally, AGD has poor diagnostic accuracy for both endometriosis and PCOS, so searching for an appropriate combination of markers to improve the accuracy is another important task for the future.

This study systematically reviewed the literature on the relationships of AGD with two gynecological disorders: endometriosis and PCOS. This review provides a comprehensive summary of the findings and methods of the included studies. The findings and methods of the included studies are somewhat heterogeneous. The review itself also has limitations. There may be other studies that were not identified, as we found only 10 eligible studies. The included studies also had a lack of patients diagnosed based on histology.

In conclusion, this review determined the relationships of AGD with two gynecological disorders: endometriosis and PCOS. AGD was longer in PCOS patients than controls, and AGD is closely related to hyperandrogenemia. In contrast, compared to controls, AGD was shorter in patients with endometriosis, especially DIE. However, further studies are needed to verify our results, which may lead to the possibility of using AGD, a simple and noninvasive measurement, as a biomarker to help to diagnose these disorders.

## Data Availability Statement

The original contributions presented in the study are included in the article/**Supplementary Material**. Further inquiries can be directed to the corresponding authors.

## Author Contributions

ZP drafted manuscript and analyzed data. FZ revised manuscript and analyzed data. KZ contributed conception and revised manuscript. All authors contributed to the article and approved the submitted version.

## Conflict of Interest

The authors declare that the research was conducted in the absence of any commercial or financial relationships that could be construed as a potential conflict of interest.

## References

[1] Schwartz CL Christiansen S Vinggaard AM Axelstad M Hass U Svingen T. Anogenital Distance as a Toxicological or Clinical Marker for Fetal Androgen Action and Risk for Reproductive Disorders. Arch Toxicol (2019) 93(2):253–72. 10.1007/s00204-018-2350-5 30430187

[2] Welsh M Saunders PT Fisken M Scott HM Hutchison GR Smith LB. Identification in Rats of a Programming Window for Reproductive Tract Masculinization, Disruption of Which Leads to Hypospadias and Cryptorchidism. J Clin Invest (2008) 118(4):1479–90. 10.1172/jci34241 PMC2267017 18340380

[3] Jain VG Singal AK. Shorter Anogenital Distance Correlates With Undescended Testis: A Detailed Genital Anthropometric Analysis in Human Newborns. Hum Reprod (2013) 28(9):2343–9. 10.1093/humrep/det286 23838161

[4] Singal AK Jain VG Gazali Z Shekhawat P. Shorter Anogenital Distance Correlates With the Severity of Hypospadias in Pre-Pubertal Boys. Hum Reprod (2016) 31(7):1406–10. 10.1093/humrep/dew115 27165620

[5] Sipahi M Tokgöz VY Alanya Tosun Ş. An Appropriate Way to Predict Fetal Gender at First Trimester: Anogenital Distance. J Matern Fetal Neonatal Med (2019) 32(12):2012–6. 10.1080/14767058.2018.1424131 29298531

[6] Dean A Sharpe RM. Anogenital Distance or Digit Length Ratio as Measures of Fetal Androgen Exposure: Relationship to Male Reproductive Development and Its Disorders. J Clin Endocrinol Metab (2013a) 98(6):2230–8. 10.1210/jc.2012-4057 23569219

[7] Castaño-Vinyals G Carrasco E Lorente JA Sabaté Y Cirac-Claveras J Pollán M. Anogenital Distance and the Risk of Prostate Cancer. BJU Int (2012) 110(11 Pt B):E707–10. 10.1111/j.1464-410X.2012.11516.x 22984847

[8] Dean A Sharpe RM. Clinical Review: Anogenital Distance or Digit Length Ratio as Measures of Fetal Androgen Exposure: Relationship to Male Reproductive Development and Its Disorders. J Clin Endocrinol Metab (2013b) 98(6):2230–8. 10.1210/jc.2012-4057 23569219

[9] Hua XG Hu R Hu CY Li FL Jiang W Zhang XJ. Associations Between Hypospadias, Cryptorchidism and Anogenital Distance: Systematic Review and Meta-Analysis. Andrologia (2018) 50(10):e13152. 10.1111/and.13152 30251425

[10] Zondervan KT Becker CM Koga K Missmer SA Taylor RN Viganò P. Endometriosis. Nat Rev Dis Primers (2018) 4(1):9. 10.1038/s41572-018-0008-5 30026507

[11] Nnoaham KE Hummelshoj L Webster P d'Hooghe T de Cicco Nardone F de Cicco Nardone C. Impact of Endometriosis on Quality of Life and Work Productivity: A Multicenter Study Across Ten Countries. Fertil Steril (2011) 96(2):366–73.e368. 10.1016/j.fertnstert.2011.05.090 21718982 PMC3679489

[12] Exacoustos C Manganaro L Zupi E. Imaging for the Evaluation of Endometriosis and Adenomyosis. Best Pract Res Clin Obstet Gynaecol (2014) 28(5):655–81. 10.1016/j.bpobgyn.2014.04.010 24861247

[13] Dinsdale N Nepomnaschy P Crespi B. The Evolutionary Biology of Endometriosis. Evol Med Public Health (2021) 9(1):174–91. 10.1093/emph/eoab008 PMC8030264 33854783

[14] García-Peñarrubia P Ruiz-Alcaraz AJ Martínez-Esparza M Marín P Machado-Linde F. Hypothetical Roadmap Towards Endometriosis: Prenatal Endocrine-Disrupting Chemical Pollutant Exposure, Anogenital Distance, Gut-Genital Microbiota and Subclinical Infections. Hum Reprod Update (2020) 26(2):214–46. 10.1093/humupd/dmz044 32108227

[15] Azziz R Carmina E Chen Z Dunaif A Laven JS Legro RS. Polycystic Ovary Syndrome. Nat Rev Dis Primers (2016) 2:16057. 10.1038/nrdp.2016.57 27510637

[16] Sullivan SD Moenter SM. Prenatal Androgens Alter GABAergic Drive to Gonadotropin-Releasing Hormone Neurons: Implications for a Common Fertility Disorder. Proc Natl Acad Sci U S A (2004) 101(18):7129–34. 10.1073/pnas.0308058101 PMC406477 15096602

[17] Recabarren SE Padmanabhan V Codner E Lobos A Durán C Vidal M. Postnatal Developmental Consequences of Altered Insulin Sensitivity in Female Sheep Treated Prenatally With Testosterone. Am J Physiol Endocrinol Metab (2005) 289(5):E801–806. 10.1152/ajpendo.00107.2005 16215166

[18] Merke DP Cutler GB Jr. New Ideas for Medical Treatment of Congenital Adrenal Hyperplasia. Endocrinol Metab Clin North Am (2001) 30(1):121–35. 10.1016/s0889-8529(08)70022-7 11344931

[19] Mendiola J Sánchez-Ferrer ML Jiménez-Velázquez R Cánovas-López L Hernández-Peñalver AI Corbalán-Biyang S. Endometriomas and Deep Infiltrating Endometriosis in Adulthood Are Strongly Associated With Anogenital Distance, a Biomarker for Prenatal Hormonal Environment. Hum Reprod (2016) 31(10):2377–83. 10.1093/humrep/dew163 PMC5027925 27357299

[20] Sánchez-Ferrer ML Mendiola J Jiménez-Velázquez R Cánovas-López L Corbalán-Biyang S Hernández-Peñalver AI. Investigation of Anogenital Distance as a Diagnostic Tool in Endometriosis. Reprod BioMed Online (2017b) 34(4):375–82. 10.1016/j.rbmo.2017.01.002 28109703

[21] Sánchez-Ferrer ML Mendiola J Hernández-Peñalver AI Corbalán-Biyang S Carmona-Barnosi A Prieto-Sánchez MT. Presence of Polycystic Ovary Syndrome Is Associated With Longer Anogenital Distance in Adult Mediterranean Women. Hum Reprod (2017a) 32(11):2315–23. 10.1093/humrep/dex274 29025054

[22] Sánchez-Ferrer ML Jiménez-Velázquez R Mendiola J Prieto-Sánchez MT Cánovas-López L Carmona-Barnosi A. Accuracy of Anogenital Distance and Anti-Müllerian Hormone in the Diagnosis of Endometriosis Without Surgery. Int J Gynaecol Obstet (2019) 144(1):90–6. 10.1002/ijgo.12691 30298915

[23] Crestani A Arfi A Ploteau S Breban M Boudy AS Bendifallah S. Anogenital Distance in Adult Women Is a Strong Marker of Endometriosis: Results of a Prospective Study With Laparoscopic and Histological Findings. Hum Reprod Open (2020) 2020(3):hoaa023. 10.1093/hropen/hoaa023 32529050 PMC7275635

[24] Peters HE Laeven CHC Trimbos C van de Ven PM Verhoeven MO Schats R. Anthropometric Biomarkers for Abnormal Prenatal Reproductive Hormone Exposure in Women With Mayer-Rokitanksy-Küster-Hauser Syndrome, Polycystic Ovary Syndrome, and Endometriosis. Fertil Steril (2020) 114(6):1297–305. 10.1016/j.fertnstert.2020.06.029 33036791

[25] Rotterdam ESHRE/ASRM-Sponsored PCOS Consensus Workshop Group. Revised 2003 Consensus on Diagnostic Criteria and Long-Term Health Risks Related to Polycystic Ovary Syndrome (PCOS). Hum Reprod (2004) 19(1):41–7. 10.1093/humrep/deh098 14688154

[26] Wu Y Zhong G Chen S Zheng C Liao D Xie M. Polycystic Ovary Syndrome Is Associated With Anogenital Distance, a Marker of Prenatal Androgen Exposure. Hum Reprod (2017) 32(4):937–43. 10.1093/humrep/dex042 28333243

[27] Hernández-Peñalver AI Sánchez-Ferrer ML Mendiola J Adoamnei E Prieto-Sánchez MT Corbalán-Biyang S. Assessment of Anogenital Distance as a Diagnostic Tool in Polycystic Ovary Syndrome. Reprod BioMed Online (2018) 37(6):741–9. 10.1016/j.rbmo.2018.08.020 30361047

[28] Prieto-Sánchez MT Hernández-Peñalver AI Sánchez-Ferrer ML Mendiola J Torres-Cantero AM. Anogenital Distance and Anti-Müllerian Hormone Combined Improves the Diagnosis of Polycystic Ovary Syndrome. Hum Fertil (Camb) (2020) 1–9. 10.1080/14647273.2020.1795574 32713212

[29] Simsir C Pekcan MK Aksoy RT Ecemis T Coskun B Kilic SH. The Ratio of Anterior Anogenital Distance to Posterior Anogenital Distance: A Novel-Biomarker for Polycystic Ovary Syndrome. J Chin Med Assoc (2019) 82(10):782–6. 10.1097/jcma.0000000000000150 PMC13048143 31356564

[30] Buggio L Barbara G Dridi D Ottolini F Sergenti G Facchin F. Anogenital Distance and Gynaecological Diseases: A Narrative Review. Ital J Gynaecol Obstet (2020) 32(3):200–7. 10.36129/jog.32.03.06

[31] Asghari S Valizadeh A Aghebati-Maleki L Nouri M Yousefi M. Endometriosis: Perspective, Lights, and Shadows of Etiology. BioMed Pharmacother (2018) 106:163–74. 10.1016/j.biopha.2018.06.109 29958140

[32] Sampson JA. Heterotopic or Misplaced Endometrial Tissue. Am J Obstet Gynecol (1925) 10(5):649–64. 10.1001/archsurg.1925.01120100007001

[33] Batt RE Yeh J. Müllerianosis: Four Developmental (Embryonic) Mullerian Diseases. Reprod Sci (2013) 20(9):1030–7. 10.1177/1933719112472736 23314961

[34] Zanatta A Pereira RM Rocha AM Cogliati B Baracat EC Taylor HS. The Relationship Among HOXA10, Estrogen Receptor α, Progesterone Receptor, and Progesterone Receptor B Proteins in Rectosigmoid Endometriosis: A Tissue Microarray Study. Reprod Sci (2015) 22(1):31–7. 10.1177/1933719114549846 PMC4527422 25217304

[35] Cermik D Selam B Taylor HS. Regulation of HOXA-10 Expression by Testosterone In Vitro and in the Endometrium of Patients With Polycystic Ovary Syndrome. J Clin Endocrinol Metab (2003) 88(1):238–43. 10.1210/jc.2002-021072 12519859

[36] Kolon TF Wiener JS Lewitton M Roth DR Gonzales ET Jr. Lamb DJ. Analysis of Homeobox Gene HOXA10 Mutations in Cryptorchidism. J Urol (1999) 161(1):275–80. 10.1016/S0022-5347(01)62132-3 10037424

[37] Cheng Z Wang M Xu C Pei Y Liu JC Huang H. Mutational Analysis of HOXA10 Gene in Chinese Patients With Cryptorchidism. Andrologia (2017) 49(1). 10.1111/and.12592 27108669

[38] Jiang I Yong PJ Allaire C Bedaiwy MA. Intricate Connections Between the Microbiota and Endometriosis. Int J Mol Sci (2021) 22(11):5644. 10.3390/ijms22115644 34073257 PMC8198999

[39] Barrett ES Parlett LE Swan SH. Stability of Proposed Biomarkers of Prenatal Androgen Exposure Over the Menstrual Cycle. J Dev Orig Health Dis (2015) 6(2):149–57. 10.1017/s2040174414000646 PMC5119464 25584807

[40] Domenici L Musella A Bracchi C Lecce F Schiavi MC Colagiovanni V. Comparison of Anogenital Distance and Correlation With Vulvo-Vaginal Atrophy: A Pilot Study on Premenopausal and Postmenopausal Women. J Menopausal Med (2018) 24(2):108–12. 10.6118/jmm.2018.24.2.108 PMC6127022 30202760

[41] Sánchez-Ferrer ML Arense-Gonzalo JJ Prieto-Sánchez MT Alfosea-Marhuenda E Gómez-Carrascosa I Iniesta MA. Does the Anogenital Distance Change Across Pregnancy? Reprod BioMed Online (2020) 41(3):527–33. 10.1016/j.rbmo.2020.05.009 32586732

[42] Barrett ES Hoeger KM Sathyanarayana S Abbott DH Redmon JB Nguyen RHN. Anogenital Distance in Newborn Daughters of Women With Polycystic Ovary Syndrome Indicates Fetal Testosterone Exposure. J Dev Orig Health Dis (2018) 9(3):307–14. 10.1017/s2040174417001118 PMC5997496 29310733

[43] Perlman S Toledano Y Kivilevitch Z Halevy N Rubin E Gilboa Y. Foetal Sonographic Anogenital Distance Is Longer in Polycystic Ovary Syndrome Mothers. J Clin Med (2020) 9(9):2863. 10.3390/jcm9092863 PMC7563834 32899698

[44] Mitchell RT Mungall W McKinnell C Sharpe RM Cruickshanks L Milne L. Anogenital Distance Plasticity in Adulthood: Implications for its Use as a Biomarker of Fetal Androgen Action. Endocrinology (2015) 156(1):24–31. 10.1210/en.2014-1534 25375036 PMC4272396

[45] Pakarainen T Zhang FP Mäkelä S Poutanen M Huhtaniemi I. Testosterone Replacement Therapy Induces Spermatogenesis and Partially Restores Fertility in Luteinizing Hormone Receptor Knockout Mice. Endocrinology (2005) 146(2):596–606. 10.1210/en.2004-0913 15514086

[46] Sharpe RM. Androgens and the Masculinization Programming Window: Human-Rodent Differences. Biochem Soc Trans (2020) 48(4):1725–35. 10.1042/bst20200200 PMC7458408 32779695

[47] Rosenfield RL. The Diagnosis of Polycystic Ovary Syndrome in Adolescents. Pediatrics (2015) 136(6):1154–65. 10.1542/peds.2015-1430 PMC9923583 26598450

[48] Welt CK Styrkarsdottir U Ehrmann DA Thorleifsson G Arason G Gudmundsson JA. Variants in DENND1A Are Associated With Polycystic Ovary Syndrome in Women of European Ancestry. J Clin Endocrinol Metab (2012) 97(7):E1342–7. 10.1210/jc.2011-3478 PMC3387396 22547425

[49] Cui L Zhao H Zhang B Qu Z Liu J Liang X. Genotype-Phenotype Correlations of PCOS Susceptibility SNPs Identified by GWAS in a Large Cohort of Han Chinese Women. Hum Reprod (2013) 28(2):538–44. 10.1093/humrep/des424 23208300

[50] Sánchez-Ferrer ML Prieto-Sánchez MT Moya-Jiménez C Mendiola J García-Hernández CM Carmona-Barnosi A. Anogenital Distance and Perineal Measurements of the Pelvic Organ Prolapse (POP) Quantification System. J Vis Exp (2018) 20(139):57912. 10.3791/57912 PMC6235246 30295651

[51] Mendiola J Roca M Mínguez-Alarcón L Mira-Escolano MP López-Espín JJ Barrett ES. Anogenital Distance is Related to Ovarian Follicular Number in Young Spanish Women: A Cross-Sectional Study. Environ Health (2012) 11:90. 10.1186/1476-069x-11-90 23217457 PMC3562168

[52] Wainstock T Shoham-Vardi I Sheiner E Walfisch A. Fertility and Anogenital Distance in Women. Reprod Toxicol (2017) 73:345–9. 10.1016/j.reprotox.2017.07.009 28743560

[53] Fabregues F González-Foruria I Peñarrubia J Carmona F. Ovarian Response Is Associated With Anogenital Distance in Patients Undergoing Controlled Ovarian Stimulation for IVF. Hum Reprod (2018) 33(9):1696–704. 10.1093/humrep/dey244 30016431

[54] Wainstock T Yoles I Sergienko R Walfisch A. The Association Between Anogenital Distance, Reproductive and General Health in Adult Females- a Prospective Cohort of 17 Years. Reprod Toxicol (2019) 90:77–81. 10.1016/j.reprotox.2019.08.007 31421229

